# Comparison of video laryngoscopy with direct laryngoscopy in patients with good glottic visualization: an observational study of 348 emergency intubations

**DOI:** 10.1186/cc14287

**Published:** 2015-03-16

**Authors:** Y Kato, H Okamoto, H Uchino, T Fukuoka

**Affiliations:** 1 Kurashiki Central Hospital, Kurashiki Okayama, Japan

## Introduction

Video laryngoscopy (VL) is known to improve glottic visualization and the first-attempt success rate compared with direct laryngoscopy (DL) in emergency tracheal intubations (ETIs). Since VL does not align the oral, pharyngeal, and laryngeal axes of the upper airway, it sometimes leads to failed intubation despite good glottic visualization. We tested the hypothesis that VL has a lower firstattempt success rate of ETI than DL among patients with good glottic visualization.

## Methods

We performed a prospective observational study examining ETIs at our tertiary care institution from July 2012 to June 2014. All consecutive patients who underwent ETIs in the emergency department and ICU were included. Patients under 18 years of age, intubated with VL not using C-MAC, were excluded. After each ETI effort, the operator completed a standardized data collection form. We classified glottic visualization as good (C-L grade 1 or 2), and poor (C-L grade 3 or 4). The primary outcome was the first-attempt success rate. The primary exposure was use of VL. Potential confounders of success rate examined were age, sex, primary indication of intubation, methods of intubation, and operator level of training and specialty. Among patients with good glottic visualization, we conducted a multivariable logistic regression adjusted for potential confounders.

## Results

A total of 348 patients were included. VL attained better glottic visualization than DL (92.3% vs. 82.6%, respectively: *P *< 0.001). In total, 299 patients with good glottic visualization were included in the analysis. Of these patients, 185 (61.9%) were male, median age and body mass index were 69 (interquartile range (IQR), 51 to 77) and 22 (IQR, 20 to 24) respectively. In univariate analysis, VL group had less respiratory failure (18.3% vs. 46.8%: *P *< 0.001) and included more trauma patients (21.1% vs. 7.9%: *P *< 0.001). The first-attempt success rates were similar between two groups (82.6% vs. 77.4%: *P *= 0.286). Multivariable logistic regression analysis adjusted for potential confounders showed that the success rate of VL was similar to that of DL (odds ratio, 1.17; 95% confidence interval, 0.57 to 2.39).

## Conclusion

Despite the possible poor alignment of airway, the firstattempt success rate of VL is similar to that of DL among patients with good glottic visualization.

